# Characteristics of quantitative perfusion parameters on dynamic contrast‐enhanced MRI in mammographically occult breast cancer

**DOI:** 10.1120/jacmp.v17i5.6091

**Published:** 2016-09-08

**Authors:** Jung Kyu Ryu, Sun Jung Rhee, Jeong Yoon Song, Soo Hyun Cho, Geon‐Ho Jahng

**Affiliations:** ^1^ Department of Radiology Kyung Hee University Hospital at Gandong, College of Medicine, Kyung Hee University Seoul; ^2^ Department of General Surgery Kyung Hee University Hospital at Gandong, College of Medicine, Kyung Hee University Seoul; ^3^ Department of Medicine Graduate School, Kyung Hee University Seoul Republic of Korea

**Keywords:** breast cancer, occult breast cancer, dynamic contrast‐enhanced perfusion, magnetic resonance imaging, quantitative analysis

## Abstract

The purpose of this study was to compare the characteristics of quantitative perfusion parameters obtained from dynamic contrast‐enhanced (DCE) magnetic resonance imaging (MRI) in patients with mammographically occult (MO) breast cancers and those with mammographically visible (MV) breast cancers. Quantitative parameters (AUC, $K^{\text{trans}},k_{\text{ep}},v_{e},v_{p}$, and $w_{i}$) from 13 MO breast cancers and 16 MV breast cancers were mapped after the DCE‐MRI data were acquired. Various prognostic factors, including axillary nodal status, estrogen receptor (ER), progesterone receptor (PR), Ki‐67, p53, E‐cadherin, and human epidermal growth factor receptor 2 (HER2) were obtained in each group. Fisher's exact test was used to compare any differences of the various prognostic factors between the two groups. The Mann‐Whitney *U* test was applied to compare the quantitative parameters between these two groups. Finally, Spearman's correlation was used to investigate the relationships between perfusion indices and four factors — age, tumor size, Ki‐67, and p53 — for each group. Although age, tumor size, and the prognostic factors were not statistically different between the two groups, the mean values of the quantitative parameters, except $w_{i}$ in the MV group, were higher than those in the MO group without statistical significance (p=0.219). The $k_{\text{ep}}$ value was significantly different between the two groups (p=0.048), but the other parameters were not. In the MO group, $v_{p}$ with size, $v_{e}$ with p53, and $K^{\text{trans}}$ and $v_{p}$ with Ki‐67 had significant correlations (p<0.05). However, in the MV group, only $k_{\text{ep}}$ showed significant correlation with age. The $k_{\text{ep}}$ value was only the perfusion parameter of statistical significance between MO and MV breast cancers.

PACS number(s): 87.19.U‐, 87.61.‐c

## I. INTRODUCTION

Screening for breast cancer with mammography decreases mortality from breast cancer. However, mammography has well‐recognized limitations, especially in those patients with dense fibroglandular tissue.^(1)^ Recently, other imaging modalities, including ultrasound and magnetic resonance imaging (MRI), have been used as adjunctive screening tools. The addition of screening ultrasonography (US) has increased the detection of small breast cancers at earlier stages, but the characteristics of these US‐detected cancers are not well‐known, especially their MR perfusion parameters.^(2)^

MRI has the potential to advance the diagnosis and staging of breast cancer. ^(3)^ Multiphase acquisitions of volumetric MR images before, during, and after an intravenous injection of contrast agent are routinely used in clinical cases with subtraction or fat suppression. This method has a very high sensitivity for breast cancer diagnosis,^3^, ^4^ but cannot measure perfusion‐related physiologic parameters because only a few phases are acquired.

Dynamic contrast‐enhanced (DCE) MRI also involves multiphase scans with a high temporal resolution with acquisition of more phases to monitor the alternations of T1 relaxation due to the contrast agent.^(5)^ DCE‐MRI can be used experimentally to characterize microvasculature, providing information about tumor microvessel structure and function. Therefore, DCE‐MRI can evaluate underlying physiological changes in the metrics of tumor perfusion that could serve as biomarkers for early detection or response to treatment compared to morphological evaluations.^6^, ^7^

DCE‐MRI data permits acquisition of semiquantitative perfusion indices including the uptake integral or initial area under the time signal curve (AUC), wash‐in ($w_{i}$) or peak up‐slope, washout or down‐slope, time‐to‐peak enhancement, and peak enhancement.^(8)^ Although semiquantitative parameters have the advantage of being relatively straightforward to calculate, they do not accurately reflect contrast medium concentration in the tissue of interest.^6^, ^7^ Recently, quantitative physiological or pharmacokinetic parameters from DCE‐MRI data were obtained to evaluate physiological characteristics of tumors. The parameters include the extravasation transfer constant ($K^{\text{trans}}$), back transfer constant ($k_{\text{ep}}=K^{\text{trans}}/v_{e}$), the extravascular‐extracellular space (EES) volume fraction ($v_{e}$), and the blood plasma volume fraction ($v_{p}$).^(9)^ The transendothelial transport constant ($K^{\text{trans}}$) is a time‐dependent leakage of the contrast agent from plasma space to the EES by diffusion per minute. The transfer constant of $k_{\text{ep}}$ is a rate constant of the contrast agent's reflux from the EES back to plasma space. Those quantitative parameters may be better to evaluate breast cancers than semiquantitative parameters because of evaluating underlying tumor physiology.^(10)^

Mammographically occult (MO) breast cancers are more likely to be diagnosed at an early stage as they are detected as smaller and node negative invasive cancers by screening US.^2^, ^11^ In terms of the molecular phenotype, Bae et al.^(2)^ explained that US‐detected cancers were more likely to have the luminal A phenotype than mammographically visible (MV) cancers, which are more likely to have the HER2 phenotype Dawood et al.^(12)^ found that patients with HER2‐type and basal‐like cancers had worse survival outcomes relative to those with luminal A‐type cancers. Therefore, patients with MO cancers may have better prognoses than those with MV cancers.

Previous studies looking at the morphology of MO cancers have shown relatively circumscribed benign looking masses that are commonly looked at with a lower level suspicion in terms of being malignant.^(11)^ These results suggest that further supporting information is needed to characterize and find out the nature of MO breast cancers. However, there has been no report about the difference in perfusion indices between MV and MO breast cancers until now.

The purpose of this study was to compare the characteristics of quantitative perfusion parameters obtained from DCE‐MRI in patients with MO and MV breast cancers. Also, we tried to correlate these perfusion indices with histopathologic analyses.

## II. MATERIALS AND METHODS

### A. Study population

This study was approved by the local institutional review board committee and informed consent was waived due to the retrospective nature of this study. A search of the computerized database for patients with a histopathologic diagnosis of breast cancer during March 2007 to December

2011, who had both DCE‐MRI and mammography, identified 121 patients with 139 breast cancers (96 invasive ductal carcinoma [IDC], 29 ductal carcinoma in situ [DCIS], 5 invasive lobular carcinoma, 4 mucinous carcinoma, 2 tubular carcinoma, 1 metaplastic carcinoma, 1 lobular carcinoma *in situ*, and 1 malignant phyllodes) at our institution.

All the mammographic images in the remaining 121 patients were reviewed by two breast radiologists with 2 and 9 years of experience (S.J. Rhee and J.K. Ryu, respectively) to evaluate whether or not the negative mammography report had suspicious findings in retrospect. Among these, 21 patients were selected as having MO breast cancer. An additional eight patients were excluded because of problems with MR perfusion imaging quality (six patients) and due to motion and not having breast surgery in our hospital (two patients). This yielded 13 MO breast cancers in 13 patients, which constituted our study group. The mean age of the study group was 51.2 years (range, 33–70) and the histologic size of the tumors varied from 1.0–6.5 cm (mean tumor size, 2.58 cm). Among the 13 MO group patients, 5 underwent mammography as screening exam and other 8 patients performed mammography owing to their palpable lumps. All these lesions were confirmed with US exams.

For the control group (mammographically visible breast cancer), we selected 16 patients with breast cancers who were in similar age range or had the smallest difference in age with each patient in the study group during the same period. This selection minimized the difference of breast parenchyma composition on mammography according to patient age. Also, we tried to select the patients in the similar ratio of same tumor histology of IDC, DCIS, and other cancers compared with those of MO group. All MV cancers were also confirmed with US.

### B. Mammography

Mammographic breast compositions were classified on a four‐point scale (4=extremely dense,>75%; 3=heterogeneously dense, 51%‐75%; 2=scattered fibroglandular tissue, 25%–50%; 1=almost entirely fatty, <25%) according to the American College of Radiology Breast Imaging Reporting and Data System (ACR BI‐RADS) breast density grade.^(13)^ Mammographic findings reported as Category 1 or Category 2 were included in the MO breast cancer group.

### C. MRI acquisition and processing

#### C.1 MRI data acquisition

MRI data were acquired before surgery to evaluate the staging of each breast cancer with an Achieva 3T MRI scanner (Philips Medical System, Best, The Netherlands) using a dedicated four‐channel, phase‐array, double‐breast coil with patients in the prone position. The breast MRI protocol at our institute included axial short tau inversion recovery (STIR) T2‐weighted imaging and axial T1‐weighted imaging (T1WI) without fat saturation in both breasts, axial three‐dimensional (3D) DCE perfusion T1WI in both breasts, postcontrast enhanced sagittal fat‐suppressed T1WI for both breasts, and axial fat‐suppressed T1WI for lymph node imaging.

To obtain DCE perfusion MRI data, an axial radiofrequency‐spoiled 3D fast field echo (FFE) sequence was run with the spectral adiabatic inversion recovery (SPAIR) fat suppression technique (inversion time, TI=80 ms). Imaging parameters of the DCE perfusion MRI scan were: repetition time (TR)=3.2 ms, echo time (TE)=1.3 ms, flip angle (FA)=10°, field of view (FOV)=200 mm (anterior–posterior, AP) ×342 mm (right‐left, RL), acquisition matrix=200×342, reconstruction matrix=560×560, slice thickness=4 mm, number of slices=40, a sensitivity encoding (SENSE) factor=1.5, acquisition voxel size=1×1×4 mm and reconstruction voxel size=0.62×0.62×4 mm, fold‐over direction = right‐left, turbo field echo (TFE) factor=50 with the number of TFE shots=75, and total number of dynamics=20. The temporal resolution per volume was 18.8 s and the total scan time was 6 min and 17 s. A catheter was placed within an antecubital vein delivered 0.1 mmol/kg (9–15 mL) of the gadopentetate dimeglumine, Gd‐diethylenetriamine penta‐acetic acid (DTPA) contrast agent (Magnevist, Wayne, NJ) at 2 mL/s (followed by a 20 mL saline flush) using a Spectris Solaris MR injection system power injector (Medrad, Warrendale, PA) after the acquisition of three baseline dynamic scans for the DCE study.

#### C.2 Imaging processing

To perform quantitative DCE‐MRI data analysis, the commercially available Nordic ICE software tool (Nordic Image Control and Evaluation, version 2.3.12; NordicNeuroLab, Bergen, Norway) was used. We transferred the DICOM files from the imaging acquired computer system to a personal computer and processed it to calculate the perfusion parameters. The noise threshold level was manually defined to exclude pixels from further analysis. The prebolus range was set from 1 to 3 to specify the precontrast or baseline data range because the contrast agent was injected on the 4th scan. The wash‐in range was also manually defined to specify the range of images used to estimate the initial up‐slope of contrast enhancement and bolus arrival time. Spatial smoothing and temporal smoothing, which was 4 mm isotropic kennel for both, were used to reduce image noise and spikes in the hemodynamic signal response. The signal time curve in the input images was converted to change in 1/T1 relaxation rate. The baseline T1 value for all voxels was set to a fixed value of 1300 ms, based on the data obtained from breast tissue on a 3T MRI system.^(14)^

In this study, before semiautomatic arterial input function (AIF) determination, motion correction applied using a rigid body motion correction to the time series data, which is provided by the manufacture in the preprocess tab. A semiautomatic AIF tracking algorithm was used to obtain an AIF for each patient. This algorithm was initialized by automatic detections of arteries using the cluster analysis technique provided by the software manufacturer to search for the AIF of a manually selected rectangular area in only one slice, and then the user manually selected AIF pixels after reviewing the hemodynamic curve. AIF was measured in the ipsilateral internal mammary artery or its branches when available. If the automatic detection of AIF failed in the artery, then we selected a vessel consistently identified in the scanned area closest to the tumor, which accurately reflected the contrast agent concentration of tumor vascularity.^(15)^ As several regions of interest (ROI) for AIF were drawn on the widest luminal section area, a signal intensity change curve for each ROI was graphically reviewed. If the curve of the ROI showed a rapid increase with a sharp peak concentration followed by a washout pattern, described as a typical AIF characteristic in the literature, it was included for analysis.^(16)^

To obtain semiquantitative and quantitative maps, DCE‐MRI data were deconvolved with the tissue response curves from the AIF.^(17)^ After selecting the prebolus range, the last image in the dynamic series, wash‐in range, and AIF, we specified output maps for the conventional curve parameters for the DCE‐MRI data, which were AUC and $w_{i}$. In addition, for the pharmacokinetic parameters, maps of $K^{\text{trans}},k_{\text{ep}},v_{e}$, and $v_{p}$ were also estimated. We used the extended Tofts model provided by the software manufacture. Freehanded volumetric ROI of the entire lesion over several slices was drawn on the AUC map using the MRIcro software (www.sph.sc.edu/comd/rorden/mricro.html) by the radiologist (J.K.R.). The mean value over the volumetric ROI for each parametric factor was obtained.

### D. Histopathologic analysis

All 29 patients underwent surgery. Histopathologic reports were reviewed, including tumor size, axillary nodal status, histologic grade, nuclear grade, estrogen receptor (ER) status, progesterone receptor (PR) status, Ki‐67, p53, c‐erbB‐2, cytokeratin5/6 (CK5/6), and E‐cadherin. ER and PR positivity were defined as the presence of 10% or more positively stained nuclei in 10 high‐power fields. The Ki‐67 labeling index was calculated as the percentage of positive tumor nuclei divided by the total number of tumor cells examined. Commonly, Ki‐67 values of 20% or more are considered high; however, we considered exact Ki‐67 values as continuous variables for statistical analysis. For evaluation of p53 and CK 5/6, positivity is typically defined as the presence of any cytoplasmic and/or membranous staining in the tumor cells. However, we applied exact p53 values in percentage as continuous variables for statistical analysis to correlate with perfusion indices.

The intensity of c‐erbB‐2 staining was scored as 0, 1+, 2+, or 3+. Tumors with a 3+ score were classified as human epidermal growth factor receptor 2 (HER2)‐positive and tumors with the 0 or 1 + score were classified as HER2‐negative. Gene amplification using fluorescence *in situ* hybridization was used to determine HER2 status in tumors with the 2+ score based on immunohistochemistry. HER2 expression was considered positive if the ratio of HER2 gene copies to chromosome 17 was >2.

### E. Statistical analyses

Fisher's exact test was used to compare group differences of acquired prognostic factors such as menopausal status, tumor histology, histologic grade, breast density, PR, ER, CK5/6, HER‐2, E‐cadherin, axillary LN metastasis, and nuclear grade. The Mann‐Whitney *U* test was used to compare differences in age, tumor size, Ki‐67, p53, and the mean values of the perfusion indices between MO and MV groups. We also compared perfusion indices between MO and MV groups in 18 patients with IDCs, 21 patients with dense breasts, 25 ER positive patients, and 20 PR positive patients in a subgroup analysis using the Mann‐Whitney *U* test.

We used the nonparametric statistical technique because the subject population was small. Finally, Spearman's correlation was used to investigate any relationships between perfusion indices and the four factors, age, tumor size, Ki‐67, and p53, for each group. Statistical analyses were performed using commercially available software (MedCalc version 12.7, Ostend, Belgium). Statistical significance was assigned if the p‐value was <0.05.

## III. RESULTS

The mean age of the control group was 48.8 years (age range, 37–64) and the histologic size of the tumors ranged from 1.5–5.0 cm (mean tumor size, 2.81 cm). There was no significant statistical difference between two groups in ages (p=0.693) and tumor sizes (p=0.367). All patients in both groups received surgery at our institute, and the median interval between MRI and surgery was 4.55 days (range, 1–19 days). The time interval between the MRI and the mammography was within 15 days for all patients.

**Figure 1:**
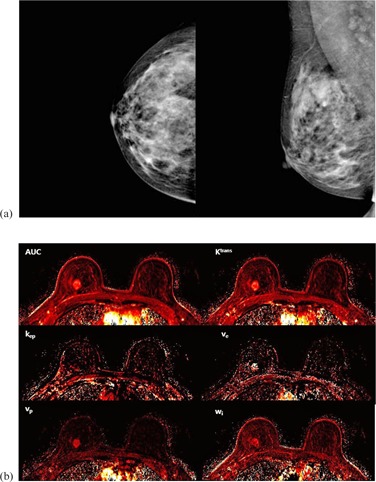
Representative case of mammographically occult (MO) group. A 37‐year‐old female with invasive ductal carcinoma in her right upper central breast: (a) craniocaudal and mediolateral oblique view. The tumor mass in right upper central breast is not visualized. (b) Perfusion maps according to each perfusion parameters.

**Figure 2:**
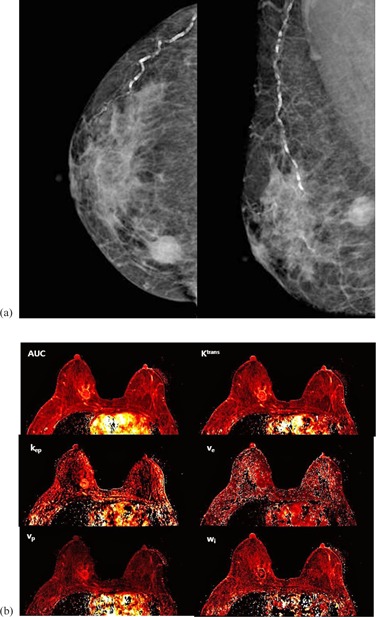
Representative case of mammographically visible (MV) group. A 53‐year‐old female with ductal carcinoma *in situ* on her right upper inner breast: (a) craniocaudal and mediolateral oblique view. An oval hyperdense mass of about 2.6 cm is easily shown in right upper inner quadrant of breast with internal clustered microcalcifications. (b) Perfusion maps according to each perfusion parameters.

The demographic characteristics and the acquired prognostic factors are summarized in Table 1. None of the differences in the prognostic factors were statistically significant between the two groups. Although Ki‐67 showed a trend (p=0.078), age, tumor size, Ki‐67, and p53 did not show statistically significant difference between the groups. Figure 1 shows an example of a case in the MO group with negative mammography and six parameter perfusion maps. Figure 2 presents a case from the MV group, in an oval mass on MG was clarified followed by perfusion maps.

Table 2 lists values (mean ± standard deviation) of the quantitative indices of DCE‐MRI data and the results of statistical analyses. The difference between the $k_{\text{ep}}$ values between the two groups was revealed to be statistically significant (p=0.048), but this was not observed in the other parameters (p>0.2). All perfusion indices values, except $w_{i}$, were higher in the MV group than those in the MO group, but the differences showed no statistical significance, except for $k_{\text{ep}}$.

For the subgroup analyses, 20 PR positive patients were composed with 11 MV patients and 9 MO patients. Only the $w_{i}$ parameter was statistically significant difference between MV and MO groups (p=0.038). The mean values of $w_{i}$ were 0.09 in MV, but 0.366 in MO. Among 18 IDC patients, there were 11 MV patients and 7 MO patients. With 21 dense breasts, there were 11 MV patients and 10 MO patients. With 25 ER positive patients, there were 15 MV patients and 10 MO patients. These subgroup analyses showed no statistically significant difference of perfusion parameters between MV and MO groups (p>0.05).

**Table 1:** Clinical and imaging characteristics of patients in the mammographically occult (MO) and the mammographically visible (MV) groups.

*Characteristics*	*MO Group*	*MV Group*	p*‐value*
Age (years)	51.15±9.44	49.81±8.04	0.693^c^
Size (cm)	2.58±1.60	2.80±1.11	0.367^c^
Menopausal status			
Premenopausal	8(61.5)	8(50.0)	0.806
Postmenopausal	5(38.5)	8(50.0)	
Family history or personal history of breast cancer	1(100.0)	0(0.0)	‐
Breast density			
Nondense (grade 1 and 2)	3(23.1)	5(31.3)	0.697
Dense (grade 3 and 4)	10(76.9)	11(68.8)	
Final BI‐RADS category			
1 and 2	13(100.0)	0(0.0)	0.001
0, 4 and 5	0(0.0)	16(100.0)	
Tumor histology			
DCIS	4(30.8)	3(18.8)	0.694
IDC	7(53.8)	11(68.8)	
Others^a^	2(15.4)	2(12.5)	
Axillary LN Metastasis			
Negative	12(92.3)	12(75)	0.343
Positive	1(7.7)	4(25)	
Histologic grade^b^			
1 or 2	6	6(46.1)	0.633
3	2(22.2)	5(38.5)	
Not Applicable	5	5	
Black'sNuclear grade			
1 or 2	8	6	0.400
3	3	6	
Not Applicable	2	4	
ER			
Negative	3(23.1)	1(6.2)	0.299
Positive	10(76.9)	15(93.8)	
PR			
Negative	4(30.8)	5(31.3)	1.000
Positive	9(69.2)	11(68.7)	
Ki‐67	17.92(±21.41)	25.25(±16.76)	0.078^c^
P53	17.31(±30.56)	21.63(±27.88)	0.597^c^
CK 5/6			
Negative	12	11	0.183
Positive	1	5	
HER2			
Negative	9	12	1.000
Positive	4	4	
E‐cadherin			
Preserved	9	12	1.000
Not preserved	4(25)	4(31)	

^a^ Others: tubular carcinoma (1), mucinous carcinoma (2), invasive lobular carcinoma (1).

^b^ Histologic grade: Modified Bloom & Richardson's histologic grade

^c^ Mann‐Whitney *U* test.

DCIS = ductal carcinoma in site; IDC = invasive ductal carcinoma

**Figure 3:**
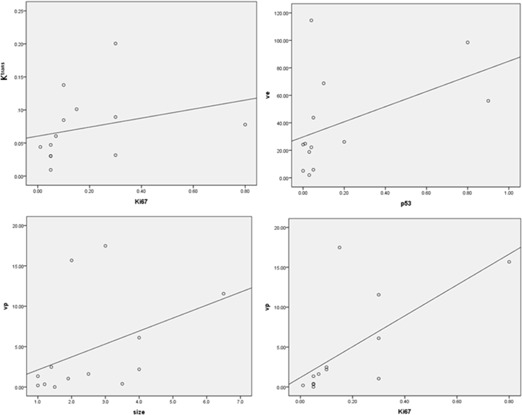
Correlation graphs between the perfusion indices and the characteristic parameters in the mammographically occult group: (top left) $K^{\text{trans}}$ and Ki‐67; (top right) $v_{e}$ and p53; (bottom left) $v_{p}$ and tumor size; (bottom right) $v_{p}$ and Ki‐67.

**Figure 4:**
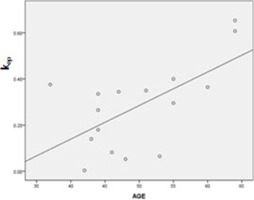
Correlation graph between the perfusion indices and the characteristic parameters in the mammographically visible group: $k_{\text{ep}}$ and age.

Results of the correlation analyses of perfusion indices with age, tumor size, p53, and Ki‐67 are listed in Table 3 for the MO group and in Table 4 for the MV group with correlation graphs, Fig. 3 and Fig. 4, respectively. In the MO group, $v_{p}$ was positively correlated with size (correlation coefficient, 0.562,p=0.046) and Ki‐67 (correlation coefficient, 0.800,p=0.001). $v_{e}$ was also positively correlated with P53 (correlation coefficient, 0.605,p=0.028), and $K^{\text{trans}}$ was positively correlated with Ki‐67 (correlation coefficient, 0.614,p=0.025). In the MV group, only $k_{\text{ep}}$ had a positive correlation with the patient's age (correlation coefficient, 0.521,p=0.038).

**Table 2:** Values (mean ± standard deviation) of the quantitative indices of dynamic contrast‐enhanced (DCE) magnetic resonance imaging (MRI) data and results of statistical analyses between the mammographically occult (MO) and the mammographically visible (MV) groups.

	*MO Group*	*MV Group*	*p‐value* ^a^
AUC	0.237±0.171	0.246±0.187	0.965
$K^{\text{trans}}$ (1/min)	0.073±0.052	0.128±0.131	0.380
k_ep_ (1/min)	0.154±0.155	0.282±0.188	0.048
v_e_ (%)	39.277±35.748	43.623±29.189	0.357
v_p_ (%)	4.645±6.180	4.876±6.561	0.483
$w_{i}$ (1/min)	0.346±0.422	0.266±0.444	0.219

^a^ Mann‐Whitney *U* test.

AUC = area under the curve.

**Table 3:** Results of correlation analyses of perfusion indices of dynamic contrast‐enhanced (DCE) magnetic resonance imaging (MRI) data with age, tumor size, P53, and Ki67 in the mammographically occult (MO) group.

		*Age*	*Size*	*P53*	*Ki67*
AUC	rho	0.300	0.220	−0.631	0.508
	*p*‐value^a^	0.320	0.469	0.84	0.076
$K^{\text{trans}}$	rho	0.410	0.416	0.547	0.614
	*p*‐value^a^	0.164	0.157	0.053	0.025
$k_{\text{ep}}$	rho	0.135	−0.284	0.494	−0.236
	*p*‐value^a^	0.661	0.347	0.086	0.438
v_e_	rho	0.237	0.529	0.605	0.278
	*p*‐value^a^	0.436	0.063	0.028	0.358
$v_{p}$	rho	0.380	0.562	0.052	0.800
	*p*‐value^a^	0.201	0.046	0.865	0.001
$w_{i}$	rho	0.539	0.259	0.052	0.800
	*p*‐value^a^	0.201	0.046	0.865	0.001

^a^ Spearman's correlation used.

AUC = area under the curve.

**Table 4:** Results of correlation analyses of perfusion indices of dynamic contrast‐enhanced (DCE) magnetic resonance imaging (MRI) data with age, tumor size, P53, and Ki 67 in the mammographically visible (MV) group.

		*Age*	*Size*	*P53*	*Ki67*
AUC	rho	0.112	−0.039	0.070	0.137
	*p*‐value^a^	0.679	0.887	0.798	0.613
K^trans^	rho	0.386	−0.107	−0.033	0.201
	*p*‐value^a^	0.140	0.694	0.905	0.456
$k_{\text{ep}}$	rho	0.521	−0.339	0.132	0.025
	*p*‐value^a^	0.038	0.126	0.627	0.926
v_e_	rho	0.239	0.179	0.175	0.377
	*p*‐value^a^	0.372	0.506	0.518	0.151
v_p_	rho	−0.066	0.159	0.006	0.174
	*p*‐value^a^	0.807	0.557	0.983	0.519
$w_{i}$	rho	−0.249	0.360	0.155	0.239
	*p*‐value^a^	0.353	0.171	0.567	0.372

^a^ Spearman correlation used.

AUC =, area under the curve.

## IV. DISCUSSION

In this study, we attempted to investigate the characteristics of quantitative perfusion parameters obtained from DCE‐MRI in patients with MO breast cancers and MV breast cancers. The $k_{\text{ep}}$ value was the most sensitive parameter to be a noticeable factor between the two groups. Furthermore, perfusion indices in the MO cancers were more highly correlated to the prognostic factors compared with those in the MV cancers. The AUC semiquantitative parameter of the DCE‐MRI model did not adequately describe the relevant physiology. This was the first study to do so, despite an earlier US‐based study.

### A. $K_{\text{ep}}$ can be distinguishing factor between MO and MV breast cancers

The $k_{\text{ep}}$, value was significantly lower in the MO group, statistically. $k_{\text{ep}}$ is the efflux rate constant from EES to plasma and $K^{\text{trans}}$ is the time‐dependent leakage constant of the contrast agent from the plasma space to EES. Therefore, both parameters are implicated in vascular permeability related with angiogenesis.

Previous studies showed that fast‐growing tumors showed higher vascular permeability and capillary density on MRI and histopathology, respectively, compared to the slow‐growing tumor model,^(18)^ because highly vascularized tumors have increased levels of vascular endothelial growth factor (VEGF).^(19)^ Although both $k_{\text{ep}}$ and $K^{\text{trans}}$ reflected vascular permeability, $k_{\text{ep}}$ was presently more sensitive than $K^{\text{trans}}$ in distinguishing MO cancers from MV cancers. It is likely that $k_{\text{ep}}$ more accurately reflects the microvascular environment, including actual tumor capillary permeability, compared to $K^{\text{trans}}$. $k_{\text{ep}}$ is only affected by the contrast concentration and fractional volumes in the tumor EES.^(20)^ However, $K^{\text{trans}}$ is influenced by blood perfusion including cardiac output, hypertension, and circulatory system of an individual, which can produce greater individual difference. Therefore, $k_{\text{ep}}$ is less dependent on other physiological variations, such as perfusion and cardiac output, which likely explains the difference in the $k_{\text{ep}}$ between the two groups, but not by $K^{\text{trans}}$. $k_{\text{ep}}$ is reportedly much more sensitive than $K^{\text{trans}}$ and $v_{e}$ in evaluating the response of chemotherapy in breast cancer patients.^(21)^ Another previous study with a mouse xenograft model also showed significant correlations of $k_{\text{ep}}$ with the initial tumor volume and changes in tumor volume.^(22)^ In addition, the previous study showed that MO cancer was smaller in size than MV cancer, but that was not the case in our study.^(5)^

Even though there was no statistically significant difference of the histologic grade between the two groups in our study, a prior study showed a higher $k_{\text{ep}}$ in patients with breast cancer who had a high histologic grade, high nuclear grade, ER negativity, and a triple‐negative subtype.^(14)^ Another previous study showed that the mean $k_{\text{ep}}$ was significantly higher in tumors showing initial rapid enhancement and a delayed washout pattern, and tumors with a high nuclear grade.^(23)^ MO cancer was detected at a relatively earlier stage compared to the MV cancer,^(11)^ was also more likely to have the luminal A phenotype than the MV cancer, and was associated with a good prognosis.^2^, ^5^, ^9^ Therefore, the perfusion parameters of $K^{\text{trans}}$ and $k_{\text{ep}}$ in the MO cancers might result in lower values than those of the MV cancers as we have hypothesized. It could be assumed that the perfusion parameters of the MO cancers reveal their inherent hemodynamic characteristics. So, these results could help to predict their features and prognosis.

### B. Perfusion indices were correlated to p53 and Ki‐67 in the MO group

Perfusion indices in the MO cancers were more highly correlated to the prognostic factors compared with those in the MV cancers. In the MO group, Ki‐67 was positively correlated with $K^{\text{trans}}\;(p=0.025)$ and $v_{p}\;(p=0.001)$, and p53 was also positively correlated with $v_{e}\;(p=0.028)$. However, we did not find correlations between the $k_{\text{ep}}$ and the prognostic factors in the MO group. In the MV group, only the $k_{\text{ep}}$ was positively correlated with the patient's age (p=0.038). These results might suggest that these two prognostic factors, p53 and Ki‐67, are expressed at very high levels in all patients in the MV group irrespective of the perfusion indices. In the MO group, correlations between prognostic factors perfusion factors varied.

Because Ki‐67 is a nuclear protein that is associated with cellular proliferation, it has been assayed in many studies as a prognostic and/or predictive marker in early breast cancer. A metaanalysis confirmed that high Ki‐67 expression in patients with early‐stage breast cancer confers a worse prognosis in the overall population.^(24)^ Another study reported a correlation between the perfusion parameters and various prognostic factors and also with immunohistochemical subtypes of breast cancer.^(25)^ The authors observed that a higher $K^{\text{trans}}$ was correlated with Ki‐67 positivity, and a higher $k_{\text{ep}}$ with CK5/6 negativity and Ki‐67 positivity. Therefore, the correlation of $K^{\text{trans}}$ with Ki‐67 in the MO cancers is similar to our results, suggesting the role of Ki‐67 in early breast cancer as a prognostic factor is influenced by the perfusion environment. Also, the positive correlations between $v_{p}$ and tumor size (p=0.046) and Ki‐67 values (p=0.001) are supported by a previous report that defines $v_{p}$ as the blood plasma volume per unit volume of tissue, which may be a marker of angiogenic activity in a tumor.^(26)^

The p53 protein is a DNA‐binding protein localized to the nucleus, which functions primarily by controlling the transcription of several other genes responsible for cell growth and also mediates cell cycle arrest and apoptosis.^(27)^ Previous data found that p53 overexpressing tumors demonstrate aggressive characteristics, including larger size, higher grade, and necrosis in comparison with p53 negative cases.^(28)^ On the other hand, $v_{e}$ is one of the main pharmacokinetic parameters suggesting EES volume fraction. It has already been reported that malignant tissues have larger interstitial water space (similar to $v_{e}$) and higher extracellular volumes than normal tissue.^(29)^ Another study observed that high‐grade gliomas showed higher $K^{\text{trans}}$ and higher $v_{e}$ than low‐grade gliomas and concluded that $v_{e}$ appears to be comparable with $K^{\text{trans}}$ in differentiating high‐grade gliomas from low‐grade gliomas.^(29)^ In our study, the positive correlation of $v_{e}$ with p53 in the MO cancer group concurs with previous studies for $v_{e}$ and p53 in that they both confer active proliferation properties of tumors implicated from their grades.

### C. Limitations of this study

There were several limitations in this study. First, we did not measure the longitudinal relaxation time in the subjects' breasts. To accurately obtain quantitative physiological parameters of DCE‐MRI data, the longitudinal relaxation time in breast must be acquired. We used the fixed T1 value of breast referenced from the 3T data. This may produce errors in the data. Second, the sample size was small. It was particularly hard to find and collect mammographically occult breast cancer patients for the study. Third, AIF was obtained from several arteries rather than a single place. The inaccuracy in the AIF could lead to errors in the estimated parameters. In breast MRI, it is important to select good temporal resolution and high spatial resolution with large spatial coverage. The temporal resolution of 18.8 s used in this study may be not optimal for AIF characterization, and it may miss the peak AIF and therefore yield larger parameter values, particularly of $K^{\text{trans}}$. Finally, parametric quantification of DCE‐MRI data is usually very complicated. A previous study showed importance in selecting the appropriate model to analyze the DCE‐MRI time courses obtained in patients with breast cancer.^(30)^ It is plausible that inappropriate model selection can lead to inaccuracy in the estimation of physiological parameters if the fitting model does not include the underlying physiological properties of a given breast tumor.

## V. CONCLUSIONS

Among several perfusion parameters, $k_{\text{ep}}$ is statistically significantly lower in the MO breast cancers compared with that of the MV breast cancers. As $k_{\text{ep}}$ reflects vascular permeability and angiogenesis in breast cancer, the results indicate that there are perfusion differences between MO breast cancers and MV breast cancers. Furthermore, perfusion indices in the MO cancers were more highly correlated to the prognostic factors compared with those in the MV cancers.

We could assume that several prognostic factors, such as Ki‐67 and p53, could be influenced by the perfusion environment in early breast cancer.

## ACKNOWLEDGMENTS

The authors thank Mr. Yong Sung Park and Dr. Jung Im Kim for expert assistance with statistical analysis. Contract grant sponsors: the Kyung Hee University; contract grant number KHU‐20120762, and the National Research Foundation of Korea (NRF) grant funded by the Korea government (MSIP); contract grant number 2014R1A2A2A01002728. This study was approved by the review board committee of the Kyung Hee University Hospital at Gangdong (KHNMC IRB 2012‐090).

## COPYRIGHT

This work is licensed under a Creative Commons Attribution 3.0 Unported License.

## References

[1] Lee CH , Dershaw DD , Kopans D , et al. Breast cancer screening with imaging: recommendations from the Society of Breast Imaging and the ACR on the use of mammography, breast MRI, breast ultrasound, and other technologies for the detection of clinically occult breast cancer. J Am Coll Radiol. 2010;7(1):18–27. 20129267 10.1016/j.jacr.2009.09.022

[2] Bae MS , Han W , Koo HR , et al. Characteristics of breast cancers detected by ultrasound screening in women with negative mammograms. Cancer Sci. 2011;102(10):1862–67. 21752153 10.1111/j.1349-7006.2011.02034.x

[3] Huang W , Fisher PR , Dulaimy K , Tudorica LA , O'Hea B , Button TM . Detection of breast malignancy: diagnostic MR protocol for improved specificity. Radiology. 2004;232(2):585–91. 15205478 10.1148/radiol.2322030547

[4] Liberman L , Morris EA , Lee MJ , et al. Breast lesions detected on MR imaging: features and positive predictive value. AJR American J Roentgenol. 2002;179(1):171–78. 10.2214/ajr.179.1.1790171 12076929

[5] Padhani AR . Dynamic contrast–enhanced MRI in clinical oncology: current status and future directions. J Magn Reson Imaging. 2002;16(4):407–22. 12353256 10.1002/jmri.10176

[6] Khalifa F , Soliman A , El–Baz A , et al. Models and methods for analyzing DCE–MRI: a review. Med Phys. 2014;41(12):124301. 25471985 10.1118/1.4898202

[7] Bergamino M , Bonzano L , Levrero F , Mancardi GL , Roccatagliata L . A review of technical aspects of T1‐weighted dynamic contrast–enhanced magnetic resonance imaging (DCE–MRI) in human brain tumors. Phys Med. 2014;30(6):635–43. 24793824 10.1016/j.ejmp.2014.04.005

[8] Jahng GH , Li KL , Ostergaard L , Calamante F . Perfusion magnetic resonance imaging: a comprehensive update on principles and techniques. Korean J Radiol. 2014;15(5):554–77. 25246817 10.3348/kjr.2014.15.5.554 PMC4170157

[9] Choyke PL , Dwyer AJ , Knopp MV . Functional tumor imaging with dynamic contrast–enhanced magnetic resonance imaging. J Magn Reson Imaging. 2003;17(5):509–20. 12720260 10.1002/jmri.10304

[10] El Khouli RH , Macura KJ , Kamel IR , Jacobs MA , Bluemke DA . 3‐T dynamic contrast–enhanced MRI of the breast: pharmacokinetic parameters versus conventional kinetic curve analysis. AJR Am J Roentgenol. 2011;197(6):1498–505. 22109308 10.2214/AJR.10.4665 PMC3496793

[11] Cho N , Moon WK , Chang JM , Yi A , Koo HR , Han BK . Sonographic characteristics of breast cancers detected by supplemental screening US: comparison with breast cancers seen on screening mammography. Acta Radiol. 2010;51(9):969–76. 20942730 10.3109/02841851.2010.515615

[12] Dawood S , Hu R , Homes MD , et al. Defining breast cancer prognosis based on molecular phenotypes: results from a large cohort study. Breast Cancer Res Treat. 2011;126(1):185–92. 20711652 10.1007/s10549-010-1113-7 PMC3026074

[13] American College of Radiology . ACR BI–RADS Atlas, Breast Imaging Reporting and Data System. Reston, VA: American College of Radiology; 2003.

[14] Rakow–Penner R , Daniel B , Yu H , Sawyer–Glover A , Glover GH . Relaxation times of breast tissue at 1.5T and 3T measured using IDEAL. J Magn Reson Imaging. 2006;23(1):87–91. 16315211 10.1002/jmri.20469

[15] Yankeelov TE , Gore JC . Dynamic contrast enhanced magnetic resonance imaging in oncology: theory, data acquisition, analysis, and examples. Curr Med Imaging Rev. 2009;3(2):91–107. 19829742 10.2174/157340507780619179 PMC2760951

[16] Pickup S , Zhou R , Glickson J . MRI estimation of the arterial input function in mice. Acad Radiol. 2003;10(9):963–68. 13678084 10.1016/s1076-6332(03)00291-5

[17] Murase K . Efficient method for calculating kinetic parameters using T1‐weighted dynamic contrast–enhanced magnetic resonance imaging. Magn Reson Med. 2004;51(4):858–62. 15065262 10.1002/mrm.20022

[18] van Dijke CF , Brasch RC , Roberts TP , et al. Mammary carcinoma model: correlation of macromolecular contrast–enhanced MR imaging characterizations of tumor microvasculature and histologic capillary density. Radiology. 1996;198(3):813–18. 8628876 10.1148/radiology.198.3.8628876

[19] Dvorak HF , Brown LF , Detmar M , Dvorak AM . Vascular permeability factor/vascular endothelial growth factor, microvascular hyperpermeability, and angiogenesis. Am J Pathol. 1995;146(5):1029–39. 7538264 PMC1869291

[20] Koo HR , Cho N , Song IC , et al. Correlation of perfusion parameters on dynamic contrast–enhanced MRI with prognostic factors and subtypes of breast cancers. J Magn Reson Imaging. 2012;36(1):145–51. 22392859 10.1002/jmri.23635

[21] Li X , Arlinghaus LR , Ayers GD , et al. DCE–MRI analysis methods for predicting the response of breast cancer to neoadjuvant chemotherapy: pilot study findings. Magn Reson Med. 2014;71(4):1592–602. 23661583 10.1002/mrm.24782 PMC3742614

[22] Song Y , Cho G , Suh JY , et al. Dynamic contrast–enhanced mri for monitoring antiangiogenic treatment: determination of accurate and reliable perfusion parameters in a longitudinal study of a mouse xenograft model. Korean J Radiol. 2013;14(4):589–96. 23901316 10.3348/kjr.2013.14.4.589 PMC3725353

[23] Yi B , Kang DK , Yoon D , et al. Is there any correlation between model–based perfusion parameters and model–free parameters of time–signal intensity curve on dynamic contrast enhanced MRI in breast cancer patients? Eur Radiol. 2014;24(5):1089–96. 24553785 10.1007/s00330-014-3100-6

[24] de Azambuja E , Cardoso F , de Castro G, Jr. et al. Ki–67 as prognostic marker in early breast cancer: a meta–analysis of published studies involving 12,155 patients. Br J Cancer. 2007;96(10):1504–13. 17453008 10.1038/sj.bjc.6603756 PMC2359936

[25] Kim JY , Kim SH , Kim YJ , et al. Enhancement parameters on dynamic contrast enhanced breast MRI: do they correlate with prognostic factors and subtypes of breast cancers? Magn Reson Imaging. 2015;33(1):72–80. 25179138 10.1016/j.mri.2014.08.034

[26] Harrer JU , Parker GJ , Haroon HA , et al. Comparative study of methods for determining vascular permeability and blood volume in human gliomas. J Magn Reson Imaging. 2004;20(5):748–57. 15503330 10.1002/jmri.20182

[27] Kumar V , Abbas AK , Fausto N . Robbins and Cotran pathologic basis of disease. New Delhi, India: Elsevier; 2004.

[28] Radha RK, P V, B K . Histopathology and prognostic indices of carcinoma breast with special reference to p53 marker. J Clin Diagnos Res. 2014;8(7):FC04–08. 10.7860/JCDR/2014/9114.4609 PMC4149073 25177567

[29] Jakobsen I , Lyng H , Kaalhus O , Rofstad EK . MRI of human tumor xenografts in vivo: proton relaxation times and extracellular tumor volume. Magn Reson Imaging. 1995;13(5):693–700. 8569443 10.1016/0730-725x(95)00019-d

[30] Li X , Welch EB , Chakravarthy AB , et al. Statistical comparison of dynamic contrast–enhanced MRI pharmacokinetic models in human breast cancer. Magn Reson Med. 2012;68(1):261–71. 22127821 10.1002/mrm.23205 PMC3291742

